# Supplementary material for: Structure–Activity Relationship Studies of Substitutions of Cationic Amino Acid Residues on Antimicrobial Peptides
Source: Antibiotics (Basel). 2022 Dec 23;12(1):19. doi: 10.3390/antibiotics12010019 (PMC9854868; doi:10.3390/antibiotics12010019)

Supporting Information

Structure-activity relationship studies of substitutions of cationic amino acid residues on antimicrobial peptides

Mayu Takada[1], Takahito Ito[1,2], Megumi Kurashima[1], Natsumi Matsunaga[1], Yosuke Demizu [1,2]* and Takashi Misawa[1,*]

[1]National Institute of Health Sciences, 3-25-26, Tonomachi, Kawasaki-ku, Kawasaki-shi, Kanagawa 210-9501 Japan.

[2]Graduate School of Medical Life Science, Yokohama City University, 1-7-29 Yokohama, Kanagawa 230-0045, Japan.

*Correspondence: misawa@nihs.go.jp (T. M.), demizu@nihs.go.jp (Y. D.); Tel.:+81-44-270-6580

Table of contents

・HPLC analytical method P3

・Characterization of peptides P4-8

HPLC analytical method

Column	：	Preparative column	XBridge BEH C18 OBD Prep Column 5 µm 19×250 mm（Waters）
		Analysis column	Inertsil WP300 C18 5 µM 150×3.0 mm (GL Sciences)
Conditions	：	solvent A 0.1% TFA/water, solvent B 0.1% TFA/CH_3CN	
Flow rate	：	preparation	10 mL/min
		Analysis	1.0 mL/min
Gradient	：	10−90% gradient of solvent B 30 min	

Pep **1**

H-Gly-Lys-Lys-Phe-Leu-Lys-Ser-Aib-Lys-Lys-Phe-Val-Lys-Aib-Phe-Lys-NH_2

Purity　　　：99.9%

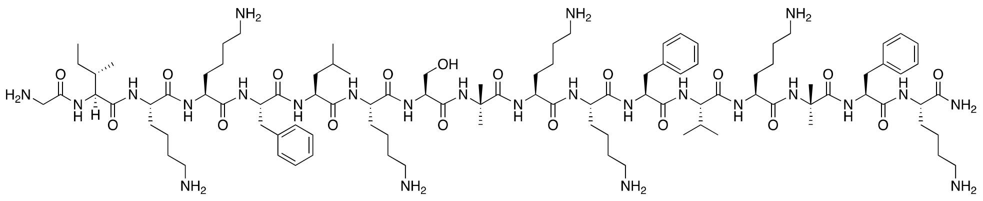

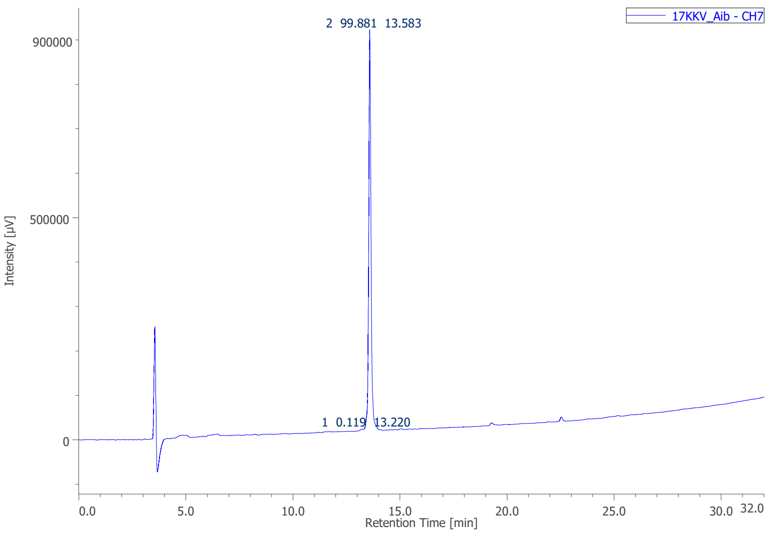

LC-MS (ESI$^+$) calcd for $C_{99}H_{167}N_{25}O_{18}[M+5H]^{5+}$: 399.85842, found: 399.8256.

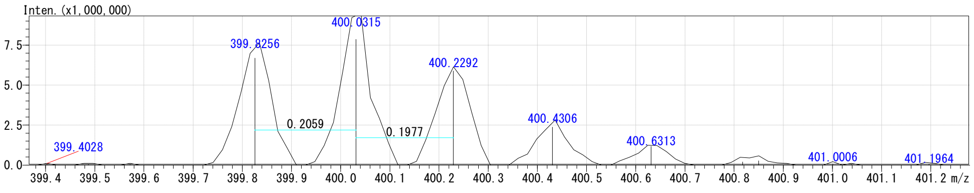

Pep **2**

H-Gly-Orn-Orn-Phe-Leu-Orn-Ser-Aib-Orn-Orn-Phe-Val-Orn-Aib-Phe-Orn-NH_2

Purity ：95.5%

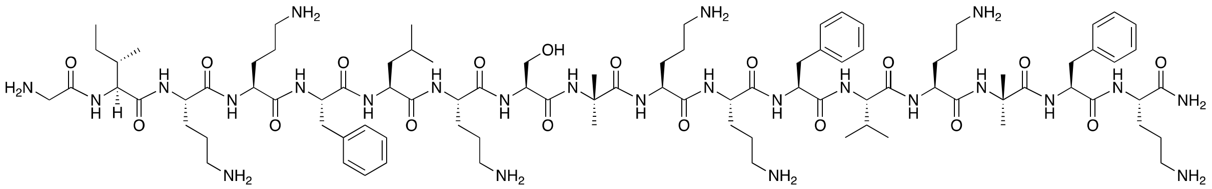

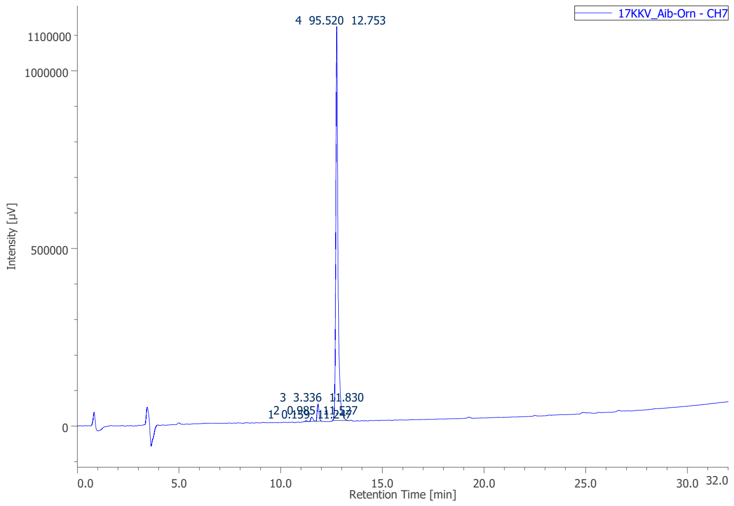

LC-MS (ESI$^+$) calcd for $C_{92}H_{153}N_{25}O_{18}[M+5H]^{5+}$: 380.2365, found: 380.2077.

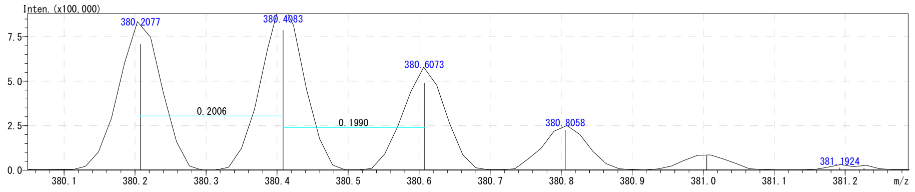

Pep **3**

H-Gly-Dab-Dab-Phe-Leu-Dab-Ser-Aib-Dab-Dab-Phe-Val-Dab-Aib-Phe-Dab-NH_2

Purity ：100.0%

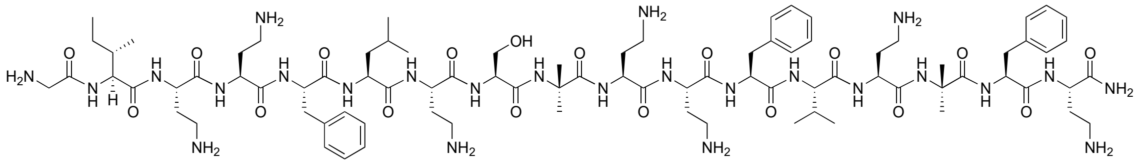

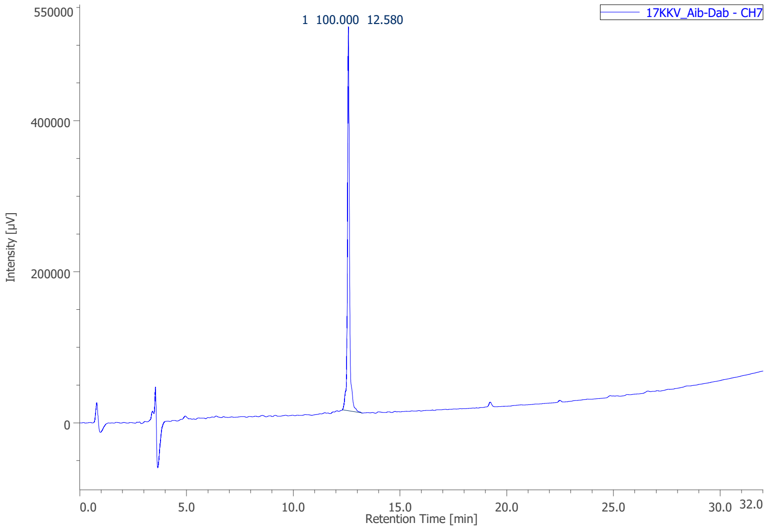

LC-MS (ESI+) calcd for $C_{85}H_{139}N_{25}O_{18}[M+5H]^{5+}$: 360.6146, found: 360.5883.

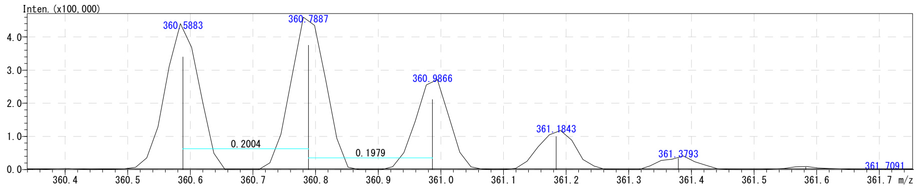

Pep **4**

H-Gly-Arg-Arg-Phe-Leu-Arg-Ser-Aib-Arg-Arg-Phe-Val-Arg-Aib-Phe-Arg-NH_2

Purity ：98.2%

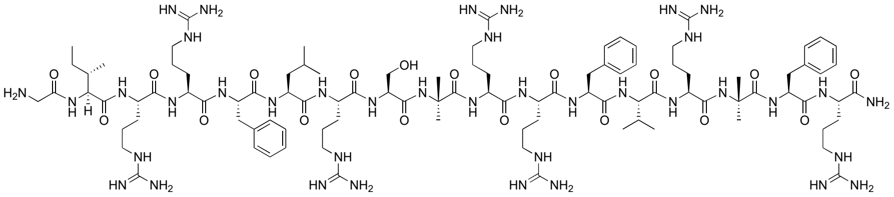

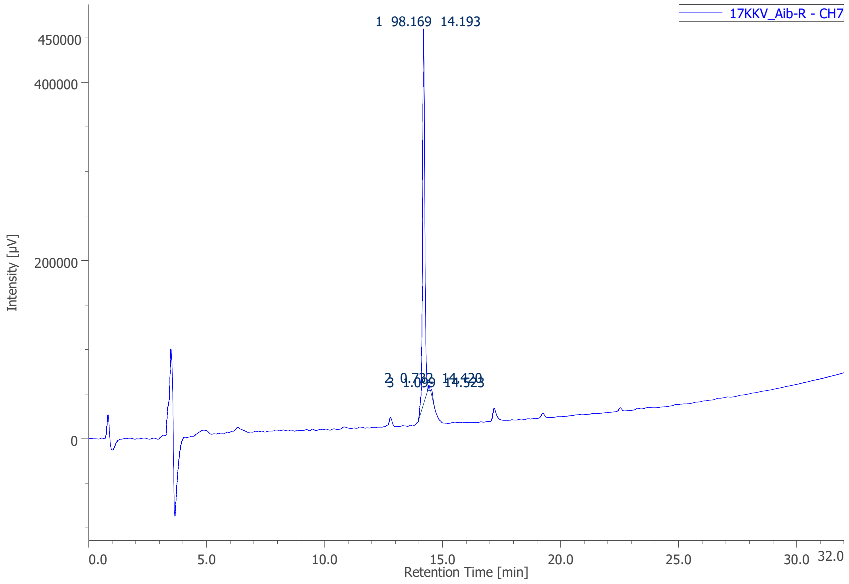

LC-MS (ESI$^+$) calcd for $C_{99}H_{167}N_{39}O_{18}[M+5H]^{5+}$: 439.06702, found: 439.0331.

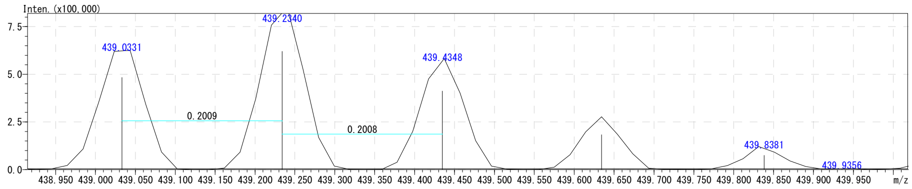

Pep **5**

H-Gly-His-His-Phe-Leu-His-Ser-Aib-His-His-Phe-Val-His-Aib-Phe-His-NH_2

Purity ：78.2%

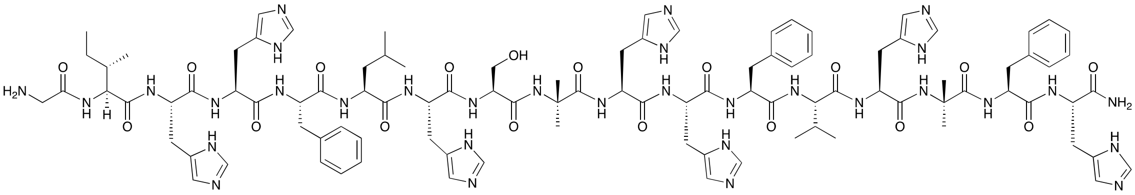

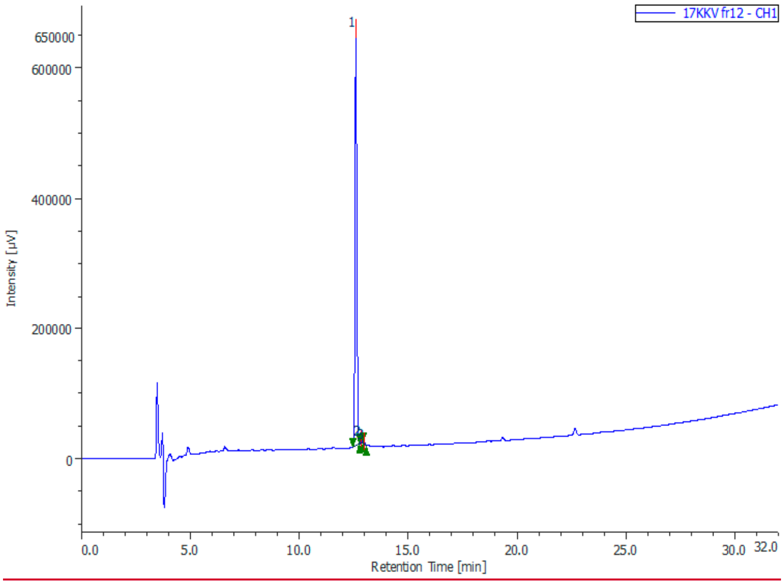

LC-MS (ESI$^+$) calcd for $C_{99}H_{132}N_{32}O_{18}[M+5H]^{5+}$: 412.40794, found: 412.3769.

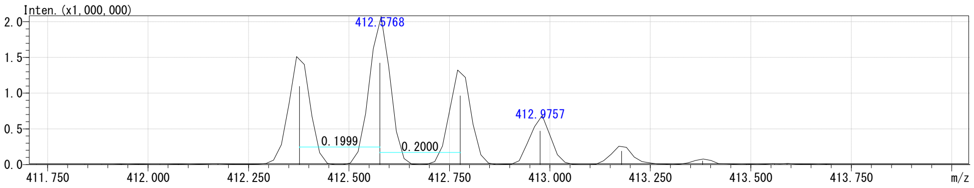

Supplement: Supplementary file 1 [file antibiotics-12-00019-s001.zip › antibiotics-2095559-supplementary.pdf]
